# Which skills are necessary for territorial pediatric nurses?

**DOI:** 10.1186/1824-7288-40-S1-A52

**Published:** 2014-08-11

**Authors:** Paolo Becherucci

**Affiliations:** 1 Pediatra di Famiglia – Vicepresidente SICuPP, Firenze, Italy

## 

Territorial primary pediatric care is provided by Family Pediatricians. According to the ACN, Family Pediatricians (PdF) may work individually, in a association or in a group (sharing the same site of the office). The new structure, in accordance with the "Balduzzi Law", instead will include two forms: The Functional Territorial Association (AFT), in which the PdF operate in their studies but are connected functionally, and the UCCP, which instead, should provide services by pediatricians, nurses and other specialists in one office. In a study conducted in Tuscany in 2010, Pediatricians resulted as being equally divided into three organizational modes. The presence of nursing personnel in the offices was almost exclusively limited to working groups. Conversely, office assistants (AdS) were present in all three categories. When the two figures worked together in the same office, the work was divided; sometimes, when there was only one type present, there was a tendency to assign one’s own work to the other person. It is therefore evident that in the prospective of radical change in regional pediatric care, the job description of the territorial pediatric nurse, what she should do in that particular job context, must be defined. Competences and a job description are the prerequisites to set a proper training process for this figure. A preventative approach to care represents one of the most important tasks of the family pediatrician. The implementation of routine health checkups (a series of regular assessments to obtain comprehensive health measures for each child) provided by the Childhood-Health project is the means for achieving this goal. The Nursing staff represents a valuable resource to assist family pediatricians in all of their activities. Nurses may do triage for accessing the study, run the self-help diagnostics (some quick tests in the office), offer vaccination counseling and perform vaccines, give advice on childcare, handle some pseudo-pathological situations and ease maternal anxiety. A document representing clear job guidelines, agreed upon by both doctors and nurses is necessary, also for legal purposes. In a time of reduced resources, there needs to be a certain degree of flexibility in the tasks assigned: obviously, for example, making appointments is a task that a nurse can do very well, but it is certainly not an "added value" for the structure in respect to the job of secretary.

